# Interplay between mobility, social participation, and symptom burden in advanced lung cancer

**DOI:** 10.1007/s00520-025-10269-3

**Published:** 2025-12-18

**Authors:** Carmine Petrasso, Joanne Bayly, Lucy Fettes, Stephen Ashford, Matthew Maddocks

**Affiliations:** 1 Cicely Saunders Institute of Palliative Care, Policy and Rehabilitation, King’s College London, Bessemer Road, SE5 9PJ London, UK; 2 York and Scarborough Teaching Hospitals NHS Foundation Trust, York, England UK; 3 Regional Hyper-acute Rehabilitation Unit, London Northwest University Healthcare NHS Trust, Regional Hyper-acute Rehabilitation Unit, London Northwest University Healthcare NHS Trust, Northwick Park Hospital, Harrow, UK

**Keywords:** Mobility, Lung cancer, Participation, Symptom burden, Palliative care

## Abstract

**Purpose:**

Advanced non-small cell lung cancer (NSCLC) is associated with a high symptom burden that negatively impacts daily functioning and quality of life. A better understanding of the time course and relationships between these constructs can help tailor rehabilitation interventions in the context of disease progression. The objectives were to describe and compare the trajectories in mobility, participation, and symptom burden and to assess associations and explore predictors of mobility change.

**Methods:**

Secondary data analysis of a longitudinal cohort study of 110 participants with NSCLC. Mobility and participation were measured using the WHODAS 2.0 subdomains, and symptom burden was measured using the POS-S. Data at baseline, 2, 4, and 6 months were analysed using repeated measures ANOVA and regression models. Change scores were compared over time, and multiple linear regression identified predictors of mobility change.

**Results:**

Overall, mobility and symptom burden remained stable, whilst participation scores showed improvement at 4 and 6 months (*p* < 0.05). Both improvements and declines in mobility status were correlated with changes in participation (*r* = 0.297–0.462, *p* < 0.001) and symptom burden (*r* = 0.151–0.368, *p* < 0.05). Baseline symptom burden was a significant predictor of mobility change, even after adjusting for demographic and clinical variables (*p* = 0.003).

**Conclusion:**

Improvements in participation despite stable mobility and symptom burden suggest an adaptive response and/or benefit from care. Mobility, related to both symptom burden and participation, offers a functional indicator that could be used to focus rehabilitation in advanced NSCLC.

## Introduction

Lung cancer remains a leading cause of cancer-related deaths globally, accounting for 18% of all cancer mortality [1]. Non-small cell lung cancer (NSCLC) is the most prevalent form, which often presents with debilitating symptoms including breathlessness, pain, and fatigue [2]. These symptoms affect both the physical and emotional well-being of individuals and frequently lead to functional decline, including reduced mobility [3, 4].

Mobility, encompassing activities such as walking, transferring, and moving through different environments, is a crucial component of functional status and a key indicator of disease progression in advanced cancer [5]. In individuals with advanced cancer, changes in mobility may serve as a key indicator of disease progression [4]. Limited mobility directly impacts activity levels, reducing individuals’ ability to engage in meaningful daily activities and participate in broader social and community contexts [5, 6].

Social participation refers to a person’s involvement in activities that promote interactions with others and engagement in community life [7]. It is essential for emotional well-being and maintaining a sense of purpose [8]. Social participation depends on factors such as time, resources, societal context, and the individual’s preferences and priorities [7, 8]. Limited mobility can significantly restrict social participation, reducing opportunities for community engagement, maintaining relationships, and accessing support networks [8]. Similarly, symptom burden affects both mobility status and social participation and encompasses symptoms such as pain, fatigue, and breathlessness [9]. These symptoms exacerbate mobility limitations and restrict opportunities for meaningful social participation [9, 10]. The interplay between mobility, participation, and symptom burden creates a complex cycle whereby reduced mobility decreases activity levels, limiting social participation, while increased symptom burden exacerbates mobility limitations, contributing to isolation and a decline in overall quality of life [11–13].

Despite the growing recognition of mobility challenges faced by individuals with advanced cancer [4], there remain important gaps in understanding how mobility, social participation, and symptom burden interact over time. Previous research suggests that changes in mobility predict subsequent disability in activities of daily living (ADLs) [14], underscoring the importance of timely interventions to mitigate further functional decline [15]. Yet the dynamic interplay between mobility, participation, and symptom burden in advanced NSCLC has not been systematically quantified in longitudinal studies. Therefore, the present study aimed to investigate changes in and associations between mobility, participation, and symptom burden among people with advanced NSCLC. Our objectives were to (i) describe and compare the trajectories in mobility, participation, and symptom burden; (ii) determine the strength and direction of associations between these constructs; and (iii) explore demographic and clinical predictors of improvement and/or decline in mobility.

## Methods

### Study design

We conducted a secondary data analysis of a prospective, longitudinal, multi-site, cohort study in England. The original study was registered on the ISRCTN registry (ISRCTN14159936) [14]. The manuscript was written in accordance with the Strengthening the Reporting of Observational Studies in Epidemiology (STROBE) guidelines for observational studies [16].

### Participants, recruitment, and data collection

The dataset comprises participants recruited between July 2020 and January 2021 across 12 centres in England, including eight acute National Health Service (NHS) trusts, three hospices, and a national charity. Inclusion criteria were adults (18 years and older) with a diagnosis of inoperable NSCLC (stage III or IV), with no exclusions based on treatment status. Participants were excluded if they had a clinician-estimated life expectancy of less than 1 month, lacked capacity to consent, or were unable to complete the survey in English. Written consent was obtained from all eligible participants, who were also informed of their right to withdraw from the study at any time without any impact on their care. Prospective data collection occurred at seven time points: baseline and then monthly for 6 months. Detailed study methods have been previously published [14].

### Measures

Mobility was assessed using the mobility sub-domain of WHODAS 2.0, which comprises five items: standing for long periods, standing up from sitting, moving around inside the home, getting out of the home, and walking a long distance [17]. Each item is rated on a five-point scale, resulting in a summary score ranging from 5 to 25, with scores of 6 or higher indicating a mobility limitation [17]. Social participation was measured using the participation sub-domain of WHODAS 2.0, which comprises eight items ranging from the ability to join in with community activities to whether the condition has impacted the family’s financial resources [17]. Each item is scored on a five-point scale with summary scores ranging from 8 to 40, with higher scores indicating greater difficulties [17].

Symptom burden was assessed using the POS-S, a tool that evaluates how symptoms have affected the individual over the previous 7 days [18]. The POS-S consists of 10 items, covering a broad spectrum of physical and psychological concerns, including pain, breathlessness, fatigue, sleep disturbance, and bowel symptoms, each rated on a scale from 0 (no effect) to 4 (overwhelming effect), with higher scores indicating a greater overall symptom burden [18].

### Statistical analyses

The group-level trajectories of mobility, participation, and symptom burden over time were assessed by repeated measures ANOVA, using baseline and scores at 2, 4, and 6 months. These time points were selected as they align with the expected progression of disease and treatment-related effects in advanced NSCLC, allowing for the identification of meaningful patterns over a clinically relevant period [19]. To investigate the relationships between changes among individuals, change scores were computed by subtracting baseline values from those observed at 2, 4, and 6 months. Linear regression analyses were performed to assess how changes in mobility predicted changes in participation and symptom burden over time. Additionally, multiple linear regression was used to explore potential demographic and clinical predictors of mobility changes, including age, gender, cancer stage, Australian Karnofsky Performance Status (AKPS), Charlson Comorbidity Index, and symptom burden. Given the likelihood of missing data being ‘not missing at random’ due to reasons such as death or worsening health, imputation methods were deemed inappropriate. Cases with missing data were excluded from the analysis. Statistical significance was set at *p* < 0.05. Analyses were performed using SPSS version 29.0.2.0 [20].

### Ethical approval

Ethical approval for the original study [14] was obtained from St Giles Research Ethics Committee (ref 19/LO/1950). No further ethical approval was required for this secondary analysis of anonymised data, as determined by the Health Research Authority decision toolkit [21].

## Results

### Participant characteristics

At baseline, 110 participants were included (66.8 ± 9.4 years, range = 41–86 years; men 59%). The median mobility score was 9 (IQR 6–13), indicating baseline mobility limitations within the cohort [17]. Baseline characteristics are shown in Table 1.

**Table 1:** Participant characteristics at baseline

Characteristics	Participants (*n* = 110)
Age mean ± SD, min–max	66.8 ± 9.4, 41–86
Sex, *n* (%) Male Female	59 (53.6) 51 (46.4)
Cancer stage, *n* (%) III IV	31 (28.2) 79 (71.8)
Treatment, *n* (%) Immunotherapy or targeted therapy Chemotherapy Radiotherapy	64 (58.2) 51 (46.3) 34 (30.9)
WHODAS 2.0 mobility subdomain, median [IQR]	9 [6–13]
WHODAS 2.0 participation subdomain, median [IQR]	15 [11–19]
POS-S, median [IQR]	7 [4–13]
Charlson Comorbidity Index score, median [IQR]	9 [7–13]
Australian Karnofsky Performance Status, median (%) [IQR]	80 [60–90]
White, *n* (%)	105 (95.5)
Education, *n* (%) Secondary College Vocational Undergraduate Postgraduate	58 (52.7) 20 (18.2) 13 (11.8) 8 (7.3) 11 (10)
Lives with: *n* (%) Alone Spouse Children Formal caregiver	39 (35.5) 64 (58.2) 5 (4.5) 2 (1.8)
Accommodation type,* n* (%) House Flat with stairs access Bungalow/flat with disabled access	69 (62.3) 10 (9.1) 31 (28.2)

**Fig. 1:**
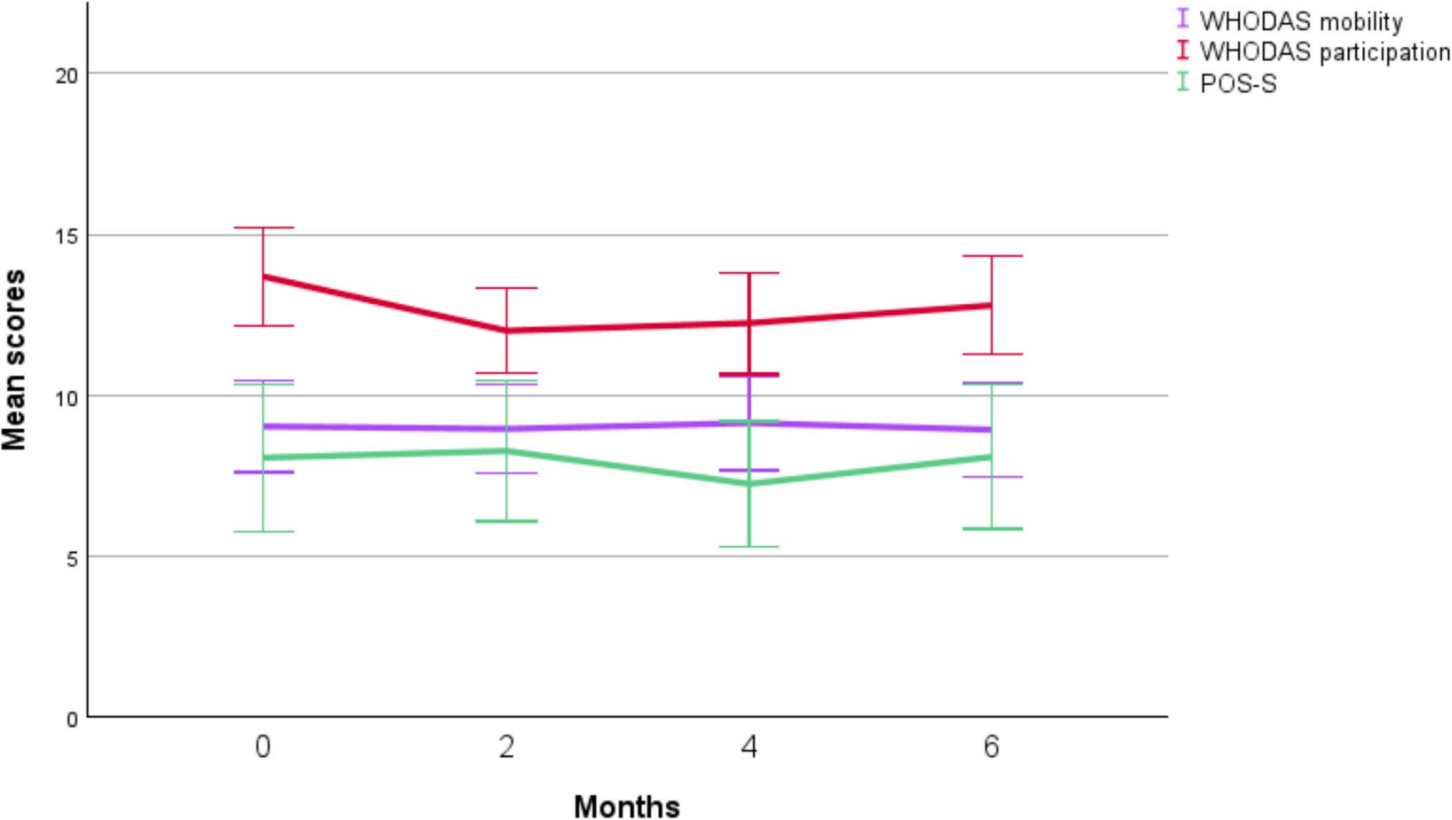
Trajectories of mobility, participation, and symptom burden presented as mean scores with 95% confidence intervals for participants with data points at baseline, 2, 4, and 6 months (*n* = 38)

Table 2 and Fig. 1 present the group-level trajectories of WHODAS mobility and participation subdomain scores, along with POS-S scores, for participants who had scores at baseline (*n* = 110), 2 (*n* = 75), 4 (*n* = 63), and 6 months (*n* = 53), and those with data at all three time points (*n* = 38). The repeated measures ANOVA results indicated that there were no statistically significant changes in the WHODAS mobility and POS-S scores (*p* > 0.05), but there were significant changes in participation scores, except between baseline and 2 months (*p* = 0.14).

**Table 2:** Comparison of changes in WHODAS 2.0 mobility and participation subdomain and POS-S (mean ± SD) scores at baseline, 2, 4, and 6 months, including participants with complete data at all time points

	WHODAS 2.0 mobility subdomain	WHODAS 2.0 participation subdomain	POS-S
**Baseline and 2 months (*n* = 75)**			
Baseline	8.88 ± 4.13	13.70 ± 4.57	7.55 ± 6.23
2 months	8.82 ± 4.16	12.70 ± 4.60	8.82 ± 6.96
*p*-value	0.89	0.14	0.11
**Baseline and 4 months (*n* = 63)**			
Baseline	9.06 ± 4.27	14.60 ± 5.28	7.81 ± 6.12
4 months	9.10 ± 5.12	11.92 ± 5.66	7.13 ± 5.78
*p-*value	0.95	< 0.001	0.35
**Baseline and 6 months (*n* = 53)**			
Baseline	8.98 ± 4.35	14.34 ± 5.42	7.68 ± 6.58
6 months	9.34 ± 4.97	12.58 ± 4.63	7.94 ± 6.68
*p*-value	0.43	0.02	0.73
**Baseline 2, 4, and 6 months (*n* = 38)**			
Baseline	9.03 ± 4.34	13.68 ± 4.65	8.05 ± 6.99
2 months	8.95 ± 4.23	12.00 ± 4.03	8.26 ± 6.67
4 months	9.13 ± 4.46	12.24 ± 4.78	7.24 ± 5.94
6 months	8.92 ± 4.45	12.79 ± 4.66	8.01 ± 6.80
*p*-value	0.96	0.04	0.53

**Fig. 2:**
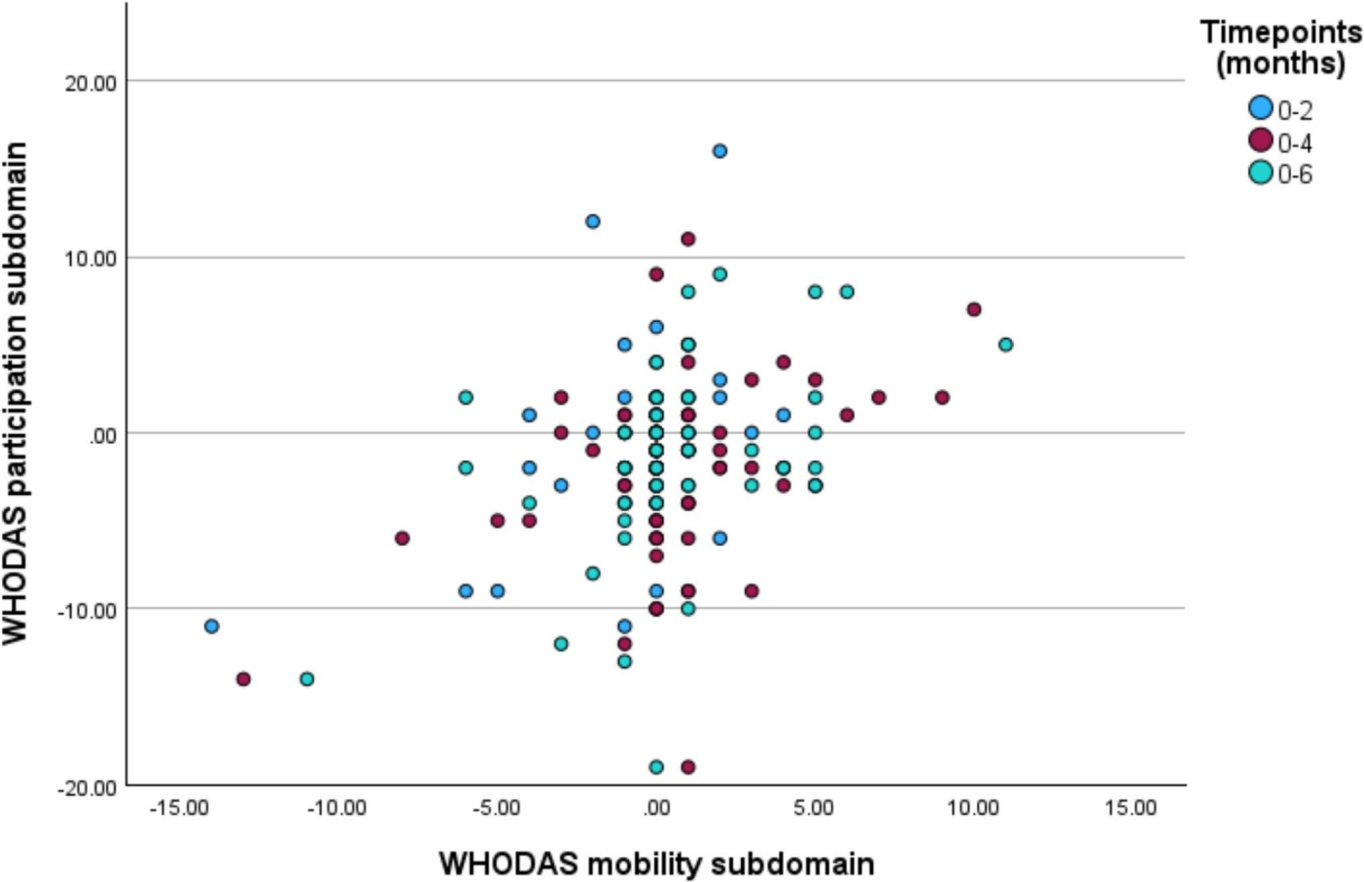
Correlation between pair-wise changes in baseline WHODAS 2.0 mobility and participation subdomain scores versus 2 (*n* = 56), 4 (*n* = 62), and 6 (*n* = 54) months

Table 3 and Fig. 2 show the regression analysis for changes in mobility and participation over time. The analysis demonstrates a positive relationship between mobility change and participation change across all time points at an individual level (*p* < 0.001).

**Table 3:** Regression analysis of changes in WHODAS 2.0 mobility and participation subdomains

	*R*	*R*^2^	Unstandardised coefficient	Significance
2 months	0.151	0.023	0.267	*p* = 0.037
4 months	0.359	0.129	0.621	*p* < 0.001
6 months	0.368	0.135	0.646	*p* < 0.001

**Fig. 3:**
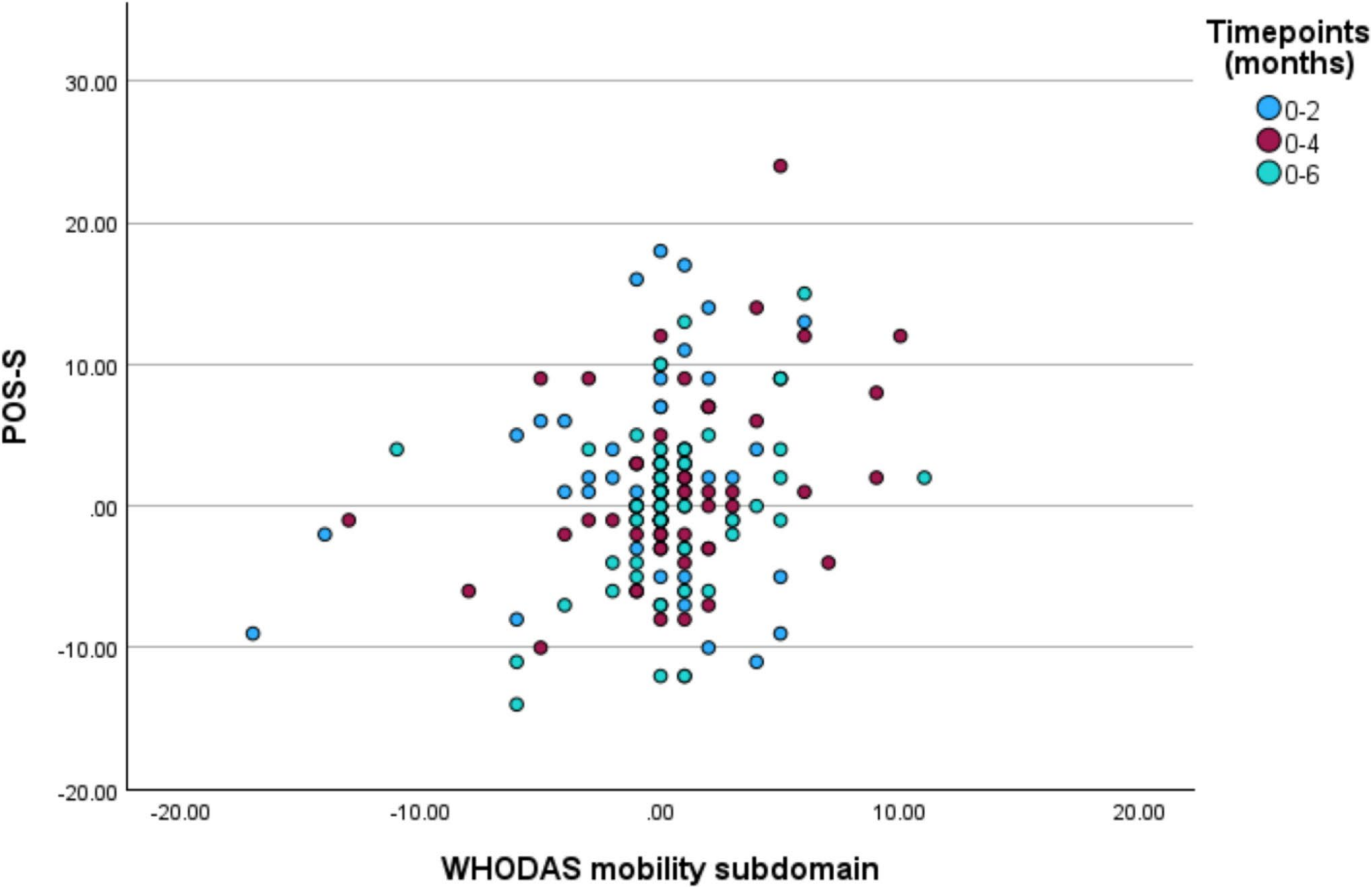
Correlation between pair-wise changes in baseline WHODAS 2.0 mobility subdomain and POS-S scores versus 2 (*n* = 64), 4 (*n* = 70), and 6 (*n* = 57) months

Table 4 and Fig. 3 illustrate the relationship between changes in mobility and symptom burden at 2, 4, and 6 months. The analysis shows a statistically significant, positive association between mobility change and symptom burden change at each time point at an individual level (*p* < 0.001).

**Table 4:** Regression analysis of changes in WHODAS 2.0 mobility subdomain and POS-scores

	*R*	*R*^2^	Unstandardised coefficient	Significance
2 months	0.297	0.088	0.531	*p* < 0.001
4 months	0.411	0.169	0.625	*p* < 0.001
6 months	0.462	0.214	0.755	*p* < 0.001

Table 5 presents the results of the multiple linear regression analysis, examining the demographic and clinical factors influencing mobility change. The model accounted for 8.9% of the variance in mobility change, indicating that the predictors explained a modest proportion of the variance in mobility change. The ANOVA test confirms that the model is statistically significant (*p* = 0.008), suggesting that these factors collectively contribute to mobility change. Among the individual predictors, symptom burden is the only statistically significant factor (*p* = 0.003).

**Table 5:** Multiple linear regression analysis for predictors of mobility change over 6 months

**Model summary**			**Value**		
*R*			0.30		
*R* square			0.089		
Adjusted *R* square			0.06		
Standard error of estimate			3.31		
**ANOVA**	**Sum of squares**	**df**	**Mean square**	**F**	***p*-value**
Regression	198.50	6	33.08	3.01	0.008
Residual	2020.41	184	10.98		
Total	2218.91	190			
**Coefficients**	**Unstandardised coefficients**	**SE**	**Standardised coefficients**	***t***	***p*-value**
Age	0.02	0.03	0.04	0.55	0.58
Gender	−0.69	0.52	−1.00	−1.34	0.18
Cancer stage	0.08	0.56	0.02	0.22	0.83
Australian Karnofsky Performance Status	0.03	0.02	0.09	1.34	0.18
Charlson Index	0.02	0.08	0.02	0.21	0.83
POS-S	0.13	0.04	0.22	3.00	0.003

Abbreviations: *df* degrees of freedom, *SE* standard error

## Discussion

This study examined the relationships between changes in mobility, participation, and symptom burden in individuals with advanced NSCLC. Our baseline sample included 110 participants who completed patient-reported measures over a 6-month period. The key findings include (i) stable group-level mobility or symptom burden across the study period, but significant changes in participation scores across most timepoints; (ii) positive relationships between individual changes in mobility and both participation and symptom burden; and (iii) symptom burden was a significant independent predictor of changes in mobility.

### Trajectories of mobility, participation, and symptom burden

Our group-level analysis revealed no significant changes in WHODAS 2.0 mobility or POS-S scores over the 6-month period, which was unexpected given the typical disease progression associated with advanced NSCLC, where a decline in mobility and an increase in symptom burden are anticipated [22]. The stability in these scores may reflect the homogeneity of our cohort, with a baseline median AKPS score of 80%, suggesting that participants, despite having stage III-IV NSCLC, were relatively stable and may have reached a functional plateau. Additionally, this finding may underestimate the true extent of mobility and symptom burden progression, as only one-third of the original sample had complete data across all time points. Individuals experiencing the most significant health deterioration were more likely to be lost to follow-up, resulting in selective attrition that may skew the results and underrepresent changes in the broader patient population.

Relative to mobility and symptom burden, participation scores showed statistically significant changes across time. While there was no significant change between baseline and 2 months (*p* = 0.14), a statistically significant improvement in participation was observed between baseline and 4 months (*p* < 0.001), with continued improvement by 6 months (*p* = 0.02). These findings indicate that although mobility and symptom burden remained stable, participants experienced fewer difficulties with engagement in daily activities, social participation, and community involvement as time progressed. The improvement in participation could suggest that as participants adapted to their illness or received more supportive care, they were able to engage more fully in life despite their cancer diagnosis [23]. This divergence between mobility and participation highlights the potential for adaptive responses even in the context of advanced disease.

### Associations between mobility, participation, and symptom burden

Despite the lack of group-level significant changes in scores over time, our findings reveal important associations between mobility, participation, and symptom burden. Changes in mobility were positively correlated with changes in participation at all time points, with the strength of this relationship increasing from *r* = 0.297 at 2 months, to *r* = 0.370 at 6 months. These findings suggest the important role of mobility in facilitating social engagement and daily activities. In turn, as mobility declines, participation in social activities may become more difficult, leading to increased isolation and decreased quality of life [24].

Similarly, the positive association between mobility and symptom burden reinforces the interconnectedness of physical and symptomatic health. As symptom burden, including pain, fatigue, and breathlessness, worsens, mobility declines, creating a feedback loop where reduced physical function exacerbates symptoms and vice versa [9]. This interrelationship suggests a synergistic dynamic where changes in mobility and symptom burden may influence one another over time. However, while our analysis identifies a temporal association, it remains uncertain whether changes in mobility drive changes in symptom burden, whether symptom burden initiates mobility decline, or if the interaction is bidirectional.

Although group-level analyses showed no significant change in mobility or symptom burden across the cohort, our individual-level analyses identified statistically significant associations between these constructs. This apparent discrepancy reflects the difference between stability at the population average and variability at the individual level. Some participants experienced deterioration in mobility while others improved, producing an overall pattern of stability but with significant within-person changes that were correlated with participation and symptom burden. These individual-level associations are clinically important because they highlight that even when population averages appear stable, patients may experience changes in function that require clinical attention. Taken together, these findings support the premise that optimising mobility positively impacts broader health outcomes in individuals with cancer and is important for maintaining independence and quality of life [25, 26]. The progressive strengthening of correlations over time suggests that interventions aimed at optimising mobility in the earlier stages of decline could have far-reaching benefits, not only for physical function but also for improving social participation and overall symptom burden.

While measurement overlap between POS-S and WHODAS 2.0 cannot be entirely excluded, the consistency of associations across timepoints and the longitudinal design identify a clinically meaningful link between symptom burden and mobility. This interpretation aligns with evidence that multidimensional symptom measures such as POS-S validly reflect change in patient status over short intervals [27]. Importantly, prior research has shown that symptoms such as pain, fatigue, and breathlessness are among the most significant contributors to disability and service use near the end of life [28]. Longitudinal analyses also demonstrate that symptom trajectories underpin variability in functional decline within groups, which cannot be captured by cross-sectional designs [29]. Accordingly, our findings extend this literature by quantifying the strength and direction of associations between symptom burden and mobility over time in advanced NSCLC, reinforcing the value of monitoring both constructs to detect risk of decline and to target rehabilitation strategies [30].

### Predictors of mobility change

Symptom burden emerged as the only significant predictor of mobility change in our regression analysis, with greater symptom burden predicting greater mobility limitations. A 10-point change in the global disability score, corresponding to a 10% minimal clinically important difference (MCID), highlights meaningful mobility changes in participants with high symptom burden [31]. For the mobility subdomain, particularly in critically ill individuals, the MCID ranges from 6 to 12% [31], meaning that a one-point change in mobility could represent a clinically meaningful shift. This finding supplements previous research showing that mobility limitations predict reduced participation in activities of daily living (ADLs) [14]. Our results add an important dimension, suggesting that greater symptom burden not only exacerbates mobility limitations, but also could serve as a trigger for rehabilitation referral to manage symptoms alongside mobility and function. Effective symptom management, including non-pharmacological and self-management strategies for refractory symptoms such as breathlessness, may help optimise mobility and improve overall quality of life [32]. For example, controlling breathlessness can enable individuals to perform essential tasks such as walking to the bathroom, underscoring the importance of integrated approaches that address symptom burden and mobility holistically [32].

Interestingly, variables such as age, gender, cancer stage, and comorbidity index were not significant predictors of mobility change. This contrasts with findings from other studies, where these factors often influence functional decline [33, 34]. However, the relatively small and homogeneous sample in this study that included participants with similar baseline functionality may have limited the ability to detect associations with these variables. Future research with more diverse and larger cohorts may clarify these relationships and identify additional predictors, such as psychosocial factors or access to support systems [35], which may in turn influence mobility.

### Implications for clinical practice

The findings of this study underscore the dynamic, bidirectional interplay between mobility, participation, and symptom burden, highlighting the reciprocal influence between these domains. This reinforces the need for rehabilitation approaches that are not only reactive to decline but also proactive in supporting recovery and adaptation. Rehabilitation programmes should be tailored to individual trajectories, providing compensatory strategies and support for those experiencing decline, while enabling individuals showing improvement or stability to maximise mobility, participation, and independence. Such programmes, adopting goal-oriented and person-centred approaches, may address mobility limitations whilst simultaneously managing symptom burden and improving social participation [15, 36].

### Strengths and limitations

A key strength of this study is its longitudinal design, with repeated measures over 6 months, providing valuable insights into functional trajectories in this population. Additionally, the use of validated self-reported measures ensured that outcomes were assessed from the individual’s perspective, capturing their lived experiences. However, some limitations also warrant consideration. Firstly, high levels of missing data, with only one-third of participants completing all time points, may have affected the stability of the trajectories observed. Secondly, the POS-S includes a mobility item, which may have contributed to the observed associations with WHODAS 2.0 mobility. This measurement overlap should be considered when interpreting the findings. Thirdly, the absence of objective physical function measures, such as performance-based tests, may have missed more granular or dynamic changes in mobility. However, evidence suggests that self-reported and performance-based measures assess the same constructs and yield comparable insights, with self-report offering a practical, reliable, and person-centred approach to capturing this type of activity over time [37]. Finally, the exclusion of non-English speaking participants and the predominantly white cohort (95.5%) limits the generalisability of findings to more diverse populations.

## Conclusion

This study suggests the stability of mobility and symptom burden at the group level in people with advanced NSCLC over 6 months. Despite the lack of significant group-level changes, individual-level associations between mobility, participation, and symptom burden emphasise their interconnectedness and the critical role of mobility in broader health outcomes. Symptom burden was identified as a key predictor of mobility change, which underscores the importance of both symptom and mobility management to optimise physical function and social participation. These findings support the need for holistic, integrative care approaches that address physical, psychological, and social dimensions of health, ultimately aiming to improve the quality of life for individuals with advanced lung cancer.

## Data Availability

No datasets were generated or analysed during the current study.
